# Geographical patterns of *Fejervarya limnocharis* gut microbiota by latitude along mainland China’s coastline

**DOI:** 10.3389/fmicb.2022.1062302

**Published:** 2022-11-17

**Authors:** Na Zhao, Zhiwei Ma, Yixin Jiang, Yingying Shi, Yuning Xie, Yuting Wang, Siyu Wu, Shelan Liu, Supen Wang

**Affiliations:** ^1^School of Ecology and Environment, Anhui Normal University, Wuhu, China; ^2^Collaborative Innovation Center of Recovery and Reconstruction of Degraded Ecosystem in Wanjiang Basin Co-founded by Anhui Province and Ministry of Education, Anhui Normal University, Wuhu, China; ^3^Provincial Key Laboratory of Biotic Environment and Ecological Safety in Anhui, Anhui Normal University, Wuhu, China; ^4^Anhui Provincial Key Laboratory of the Conservation and Exploitation of Biological Resources, College of Life Sciences, Anhui Normal University, Wuhu, China; ^5^Department of Infectious Diseases, Zhejiang Provincial Center for Disease Control and Prevention, Hangzhou, Zhejiang, China

**Keywords:** biogeographic patterns, latitude, amphibian, gut microbiota, 16S rRNA

## Abstract

The gut microbiota affects many aspects of host biology and plays key roles in the coevolutionary association with its host. Geographical gradients may play a certain role on gut microbiota variation in the natural environment. However, the distribution pattern of amphibian gut microbiota in the latitudinal gradient remains largely unexplored. Here, we sampled six natural populations of *Fejervarya limnocharis* along the eastern coastline of mainland China (spanning 20°–30° N = 1,300 km) using 16S rRNA amplicon sequencing to characterize the gut microbiota. First of all, a significant correlation between gut microbial diversity and latitude was observed in our research system. Second, we discovered that latitude influenced the composition of the gut microbiota of *F. limnocharis*. Finally, we detected that geographical distance could not determine gut microbiota composition in *F. limnocharis*. These results indicate that latitude can play an important role in shaping the gut microbial diversity of amphibian. Our study offers the first evidence that gut microbial diversity of amphibian presents a latitudinal pattern and highlights the need for increased numbers of individuals to be sampled during microbiome studies in wild populations along environmental gradients.

## Introduction

Understanding geographical distributions of gut microbiota and identifying which factors contribute to gut microbial diversity are critical steps for biodiversity conservation, because the gut microbiota affects many aspects of host health and key vital functions (such as immune function, metabolism, and inflammation) and plays key roles in the coevolutionary association with its host (Ley et al., 2008; Huang et al., 2022). Numerous studies have established that microbial communities can exhibit biogeographic patterns, and in many cases these patterns are qualitatively similar to those of macro-organisms (Sullam et al., 2012; Linnenbrink et al., 2013; Suzuki et al., 2019, 2020). However, the biogeographical pattern of microbiota is largely unclear in relative to that in macro-organisms, and the underlying mechanisms are still largely unknown (Meyer et al., 2018).

The latitudinal gradient is one of the most striking geographic gradients (Schemske et al., 2009). It shows that species diversity is negatively correlated with latitude because latitudinal spatial variation in biotic and abiotic conditions leads to changes in spatial selective pressures (Gaston, 2007). Low-latitude hosts may make high gut microbial diversity increase metagenomic diversity to increase fitness (Liu et al., 2022). High-latitudes hosts may select low gut microbial diversity in order for specific microbes to buffer the abiotic pressures (Pugnaire et al., 2019). Researchers have only begun to understand how ecological forces that drive gut microbial biogeographic patterns at macroscales (Sudakaran et al., 2012; Sullam et al., 2012; Linnenbrink et al., 2013; Ren et al., 2017; Goertz et al., 2019; Suzuki et al., 2019, 2020), while the distribution pattern of gut microbiota in the latitudinal gradient remains unresolved. Furthermore, few studies have investigated geographical patterns and ecological and evolutionary drivers of gut microbial diversity in wildlife (Ge et al., 2021; Henry and Ayroles, 2022).

Amphibians are ideal for determining the effect of latitudinal increases as a result of climate change and habitat degradation because they heavily depend on specific environmental conditions since they are poikilotherms (Parmesan, 2007). Latitudinal gradient determines some characteristics of poikilotherms (such as body size, metabolism, diet, reproduction, and behavior), and they have a wide range of physiological adaptations that vary within members of clines when the same species lives in different latitudinal gradients (Gillooly et al., 2001; Ladyman et al., 2003; Angielczyk et al., 2015; Liu et al., 2018). Mainland China’s coastline is located in Southeast Asia and spans eight different climatic zones and a distance of approximately 18,000 km (Cai et al., 2022), providing an ideal natural gradient of different environmental conditions (such as a stable climate and lack of gene flow; Wang et al., 2014). Amphibians on the coastline of mainland China allow for the study of the effect of environmental conditions on the gut microbial biota of poikilotherms along the latitudinal gradient.

**Figure 1:**
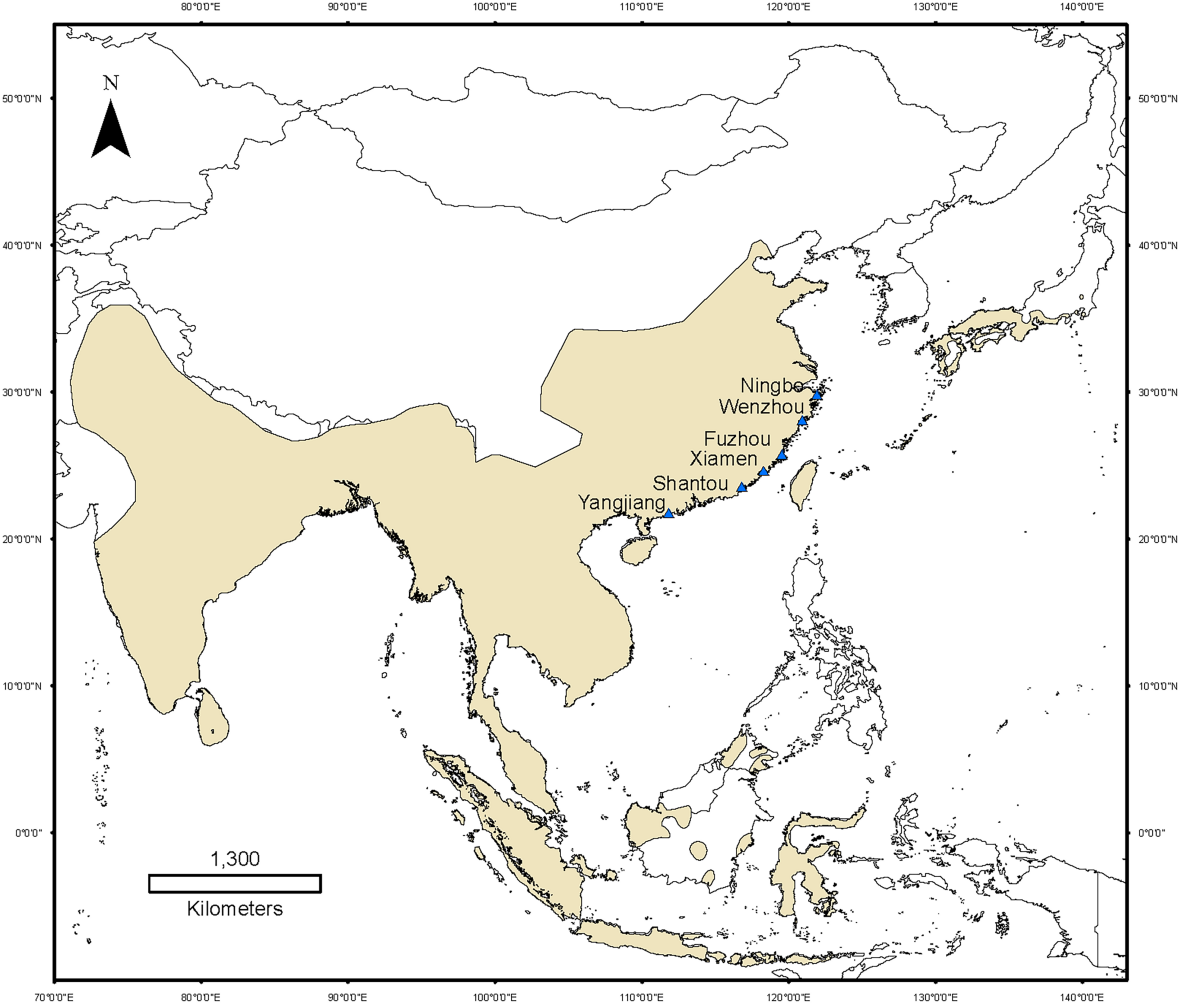
Map of *Fejervarya limnocharis* populations sampled along the eastern coast of mainland China. Yellow areas indicate the global distribution of *F. limnocharis*. Blue triangle denotes the sampling sites.

*Fejervarya limnocharis* is an excellent model to investigate evolutionary responses in clines. *Fejervarya limnocharis* is found from 10°S to 40°N along the latitudinal gradient (Figure 1), and typically located in grass, crops and stones around rice fields, dry land, ponds, bogs and ditches (Wu et al., 2006). They emerge from hibernation at the end of March to early April. Males mature in less than 1 year and are characterized by a black pigment on the throat (Li et al., 2011). Females become mature after 1 year. The maximal life span is about 35 years for males and 4–5 years for females (Li et al., 2011). *Fejervarya limnocharis* breed between the end of April and late August. New juvenile frogs after metamorphosis can be found as early as July. There are no hunting activities carried out by humans on this frog in field (Wu et al., 2006). The diet of the *F. limnocharis* is mainly composed of arthropods with no significant difference among seasons and between males and females (Li et al., 2011). Seasonal foods and body mass were significantly correlated with gut microbial diversity of *F. limnocharis* (Chang et al., 2016; Huang et al., 2017; Xu et al., 2020; Huang and Liao, 2021).

To study whether the latitudinal gradient could affect the gut microbiota of amphibians, we sampled six natural populations of *F. limnocharis* near the eastern coastline of mainland China (spanning 20°–30° N = 1,300 km) using 16S rRNA amplicon sequencing to characterize the gut microbiota. We identified latitudinal patterns of the gut microbial diversity of *F. limnocharis* along the coastline of mainland China and evaluated the effects that climate factors had on the gut microbial diversity of *F. limnocharis*.

## Materials and methods

### Ethical approval

The animal study was reviewed and approved by Animal Care and Use Committee of Anhui Normal University.

### Study sites and sampling

We conducted six line transects having a width of 2 m and a length of 100 m along the accessible edges of the rice fields, the shorelines of ponds, ditches, and dry land on each site. These were paralleled at an interval of about 15–20 m. We searched for *F. limnocharis* in transects with an LED lamp (Warsun W81, Warsun Optoelectronic Technology Company, Ningbo, China) between 19:00 and 22:30, and randomly captured 10 frogs (five adults of each sex) along transects on each site (Figure 1). A total of 60 adult’s frogs were collected in a cool box with two ice bags and immediately transported to the laboratory. The gender of all individuals was confirmed by direct observation of secondary sexual characteristics. Body mass index was calculated as the weight to the nearest 0.1 mg using an electronic balance. We treated all individuals with the single pithing method. The gut contents were collected after pithing and emptied into a sterile vial and immediately stored at −80°C. Data on mean annual temperature and annual precipitation at each sampling site were obtained from the WorldClim at a resolution of 10′ (Hijmans et al., 2005).

### DNA extraction, PCR amplification, and sequencing

The frozen aliquots of gut contents (200 mg per aliquot) were added to a 2 ml screw-cap and thawed on ice until 1.4 ml ASL buffer from the QIAamp DNA Stool Mini Kit (Qiagen, Hilden, Germany) was added. The subsequent DNA extraction steps were conducted according to the QIAamp Kit protocol. The concentration of the DNA was measured using a NanoDrop™ 2000 (Thermo Scientific). Finally, the DNA was dissolved in 200 μl sterile ddH2O and stored at −20°C until use. We amplified the V3-V4 region of the 16S rRNA genes in triplicate using primers (341F: 5’-CCTAYGGGRBGCASCAG-3′, 806R: 5’-GGACTACHVGGGTWTCTAAT-3′). The polymerase chain reaction (PCR) reactions were performed using the bio-Rad T100 gradient PCR instrument with 15 μl Phusion® High-Fidelity PCR Master Mix (New England Biolabs), 0.2 μM forward and reverse primers and 10 ng template DNA per 30 μl reaction. The PCR cycle consisted of initial denaturation at 98°C for 1 min; 30 cycles of denaturation at 98°C for 10 s, annealing at 55°C for 30 s, and extension at 72°C for 30 s; and extension at 72°C for 5 min. The PCR products were mixed with the same volume of 1 × loading buffer and were detected using 2% agarose gels. PCR products were purified using the Omega Gel&PCR Clean Up Kit (Omega Bio-Tek, USA). Sequencing was operated by Shanghai Majorbio Bio-Pharm Technology Co. Ltd. (Majorbio, Shanghai, China) operated the sequencing (Liu et al., 2021).

### Bioinformatic analyses

Paired-end reads were assigned to samples based on their unique barcode and truncated by cutting off the barcode and primer sequence. The paired-end reads were merged using VSEARCH (v2.18.0; Rognes et al., 2016) and USEARCH (v10.0.240), and raw tags were successfully spliced. Quality filtering of the raw reads was performed to remove low-quality amplicon sequences by using USEARCH. The filtered reads were then processed as unique sequences using the “minuniquesize 10” parameter in VSEARCH. Putative chimeras were discarded using silva data in VSEARCH. The UPARSE algorithm clusters the sequences with 97% similarity into operational taxonomic units (OTUs) after dereplication and discarding all singletons (Edgar, 2013). Finally, a feature OTU table can be obtained by quantifying the frequency of the feature sequences in each sample. Simultaneously, the feature sequences can be assigned taxonomy, typically at the kingdom, phylum, class, order, family, genus, and species levels, providing a dimensionality reduction perspective on the microbiota. Then, we performed a subsequent analysis of alpha and beta diversities based on this output normalized data.

### Statistical analysis

Alpha diversity indexes, which are used to analyze the complexity of bacterial diversity for a sample, were calculated and displayed using R software (Version 4.2.0), such as the observed-species index, Chao1 index, Shannon-Wiener index, and Richness index (Vegan package). Rarefaction curves of OTU richness were calculated using Vegan (Oksanen et al., 2013). To evaluate the differences among samples in terms of bacterial community complexity, we calculated the beta diversity on Bray-Curtis, Euclidean distance, Jaccard matrix, weighted UniFrac and unweighted UniFrac using Vegan, Phyloseq, Ape, GUniFrac, and Amplicon packages in R.

The variation trends of a given gut microbiota across the latitudes used Kruskal-Wallis with *post hoc* tests, and the Wilcoxon rank-sum test. Principal coordinates analysis (PCoA) and linear discriminant analysis (LDA) with different distance matrixes was used to examine the separation of species across samples. Differences in relative abundance of the microbial features were determined by LDA effect size (LEfSe; Segata et al., 2011). Analysis of functional gene content using PICRUSt2 (Douglas et al., 2020) provides proportional contributions of KEGG categories for each sample. We determined the relationships between gut microbial diversity of *F. limnocharis* and latitude, mean annual temperature, and mean annual precipitation along the eastern coast of mainland China sites using a single-variable regression. The mantel test (Vegan package) was used to analyze the correlation between differences of gut microbiota and geographical distances in different samples.

## Results

### Characteristics of the 16S rRNA gene sequence data

**Figure 2:**
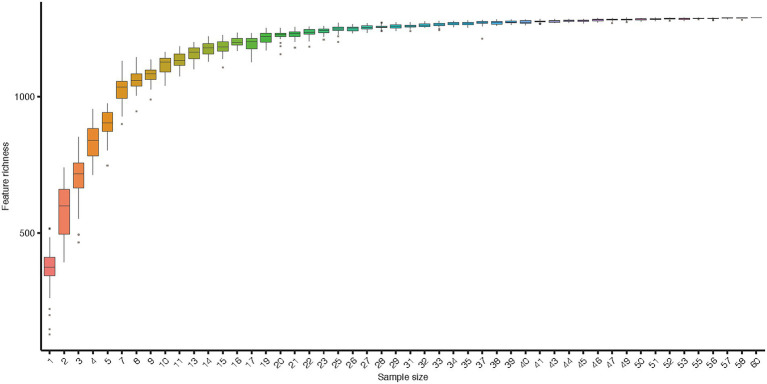
Rarefaction curve of gut microbiota. Accumulation curves for gut microbiota indices indicate that the sampling was comprehensive. Boxes denote the interquartile range (IQR) between the first and third quartiles (25th and 75th percentiles, respectively), and the line inside denotes the median. Whiskers denote the lowest and highest values within 1.5 times IQR from the first and third quartiles, respectively. Circles denote outliers beyond the whiskers.

**Figure 3:**
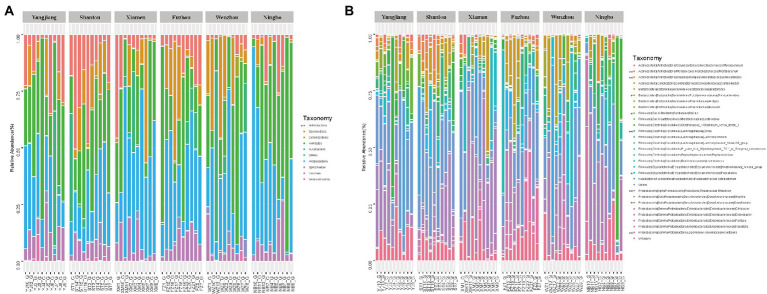
Relative contribution of gut microbiota in each sample. **(A)** Relative contribution in Phylum level. **(B)** Relative contribution in the top 30 dominant genera.

Along six coastal mainland sites (Figure 1), The V3-V4 regions of the bacterial 16S rRNA gene in gut microbiota collected from 60 adult’s frogs were sequenced to characterize the microbiotas of *F. limnocharis*. The rarefaction curves (Figure 2) approached a plateau, thereby suggesting that the number of OTUs was sufficient to reveal the authentic bacterial communities within each sample. All sequences could be classified into 18 phyla, 36 classes, 61 orders, 124 families, and 277 genera. At the phylum level (Figure 3A), Firmicutes, Proteobacteria and Bacteroidetes had the highest abundance and contributed 34.59%, 34.42%, and 11.73% of bacteria, respectively. The abundance of Actinobacteria phylum differed significantly among populations (*p* < 0.05).

At the genus level, *Desulfovibrio* (phylum: Proteobacteria), *Citrobacter* (phylum: Proteobacteria), *Peptoclostridium* (phylum: Firmicutes) and *Cetobacterium* (phylum: Fusobacteria), *Bacteroides* (phylum: Bacteroidetes) were the most abundant, and accounted for 15.46%, 6.95%, 6.39%, 6.01%, and 4.77% of bacteria, respectively (Figure 3B). The abundance of *Microbacterium*, *Nocardiopsis*, *Dorea*, *Rhizobium,* and *Rickettsiella* differed significantly among populations (wilcox test, *p* < 0.01), as did *Desulfovibrio*, *Faecalitalea*, *Bacillus,* and *Parabacteroides* (wilcox test, *p* < 0.05; Figure 3B; Supplementary Figure S1B).

### Variation of bacterial diversity of the gut microbiota in different geographic populations

**Figure 4:**
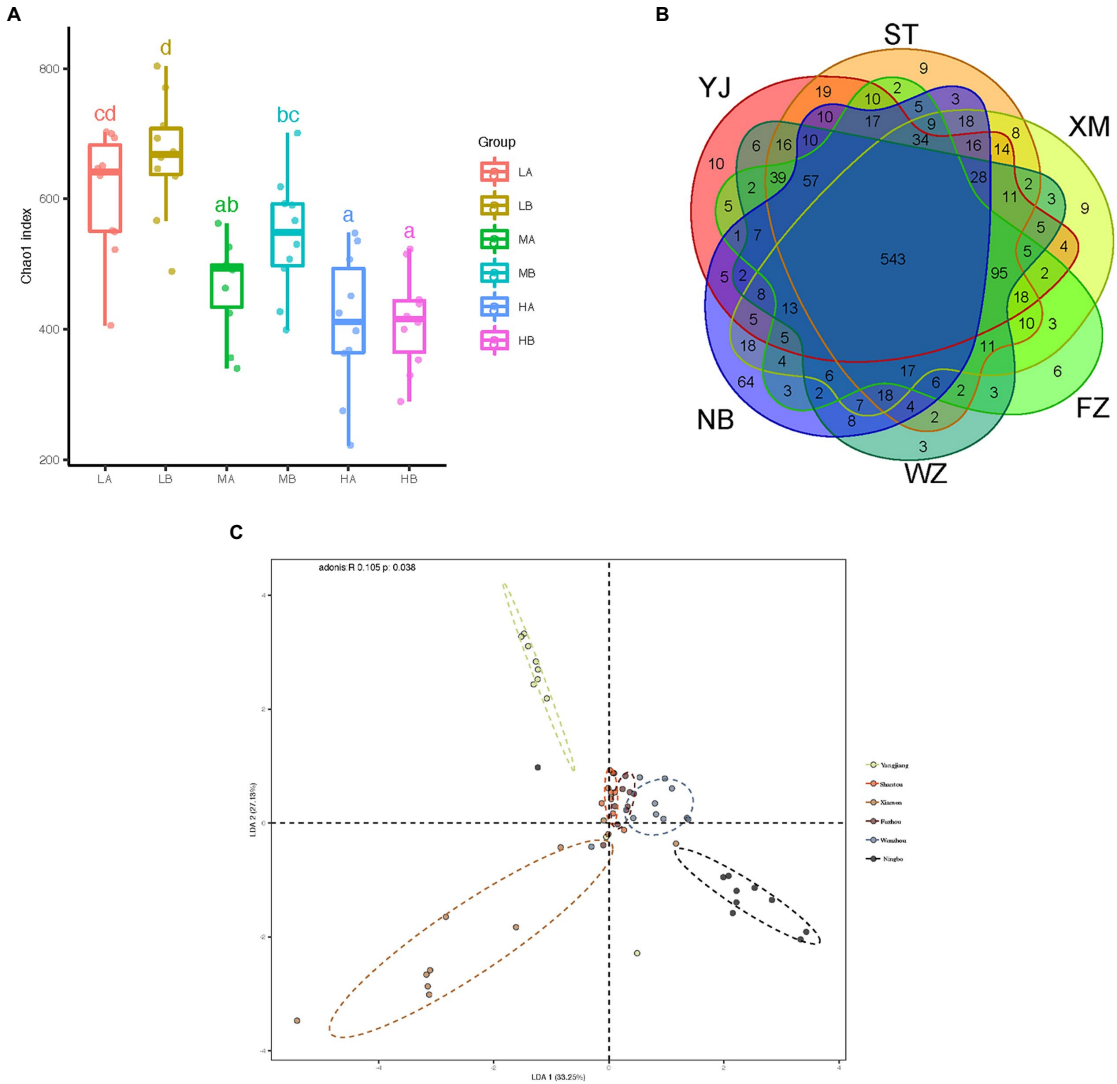
Comparison of the composition of fecal microbiota in different geographic populations. **(A)** Chao1 index of the microbiota of gut microbiota from different populations. The horizontal bars within boxes represent medians. The tops and bottoms of boxes represent the 75th and 25th percentiles, respectively. The upper and lower whiskers extend to data no more than 1.5× the interquartile range from the upper edges and lower edge of the box, respectively. **(B)** Venn diagram illustrating overlap of OTUs in gut microbiota between different groups. **(C)** The difference in the composition of the gut microbiota is illustrated by the linear discriminant analysis of the Bray-Curtis distance matrix among samples. All pairwise comparisons involved the first, second and third principal axes. The first, second and third principal axes explained most of the variances (33.25, 27.13, and 39.62%, respectively).

To further determine whether microbial communities differ among different geographic populations, we compared the diversity and richness indices among populations (Table 1). Gut microbial diversity ranged from 409.22 at Wenzhou to 665.47 at Shantou for the Chao1 index, 273 to 482.8 for the Richness index, and 3.08 to 4.58 for the Shannon index. Low latitudinal sites showed higher gut microbial diversity than high latitudinal sites (*p* < 0.05，ANOVA, Tukey-HSD test, Figure 4A). To better understand the differences in richness between different groups, the overlap of the core microbial communities between the groups was illustrated using a Venn diagram. This analysis showed that only 543 of the 1,288 OTUs accounting for the total richness were common to all of the samples (Figure 4B). These data demonstrated that approximately 57.84% of the OTUs were identified in the different geographic populations.

**Table 1:** Alpha-diversity of gut microbiota in *Fejervarya limnocharis* along the eastern coast of mainland China.

Locations	Chao1	Richness	Shannon
Yangjiang	606.01 ± 91.51	433.80 ± 88.03	4.23 ± 1.16
Shantou	665.47 ± 86.95	482.80 ± 70.80	4.58 ± 0.62
Xiamen	465.39 ± 67.82	299.10 ± 63.24	3.38 ± 0.86
Fuzhou	542.80 ± 86.06	376.70 ± 83.96	3.92 ± 0.56
Wenzhou	409.22 ± 101.91	273.00 ± 85.08	3.08 ± 0.78
Ningbo	412.53 ± 71.12	297.90 ± 66.74	3.37 ± 1.21

To measure the degree to which the gut microbiota differed in these populations, principal coordinates analysis (PCoA) was conducted based on these distance matrixes (Bray-Curtis, Jaccard, Euclidean distance, and weighted and unweighted UniFrac matrixes) between the gut samples. Although the PCoA analysis did not show a strong difference in the microbiota of individuals from different populations (Supplementary Figure S2A), the composition of the gut microbiota differed significantly from each other’s populations (all *p* < 0.01, Kruskal-Wallis test, Supplementary Figure S2B). In this seemingly contradictory situation, a linear discriminant analysis (LDA) was conducted based on the Bray-Curtis distance matrix from the gut content samples. Despite significant inter-individual variation, the gut microbiota of different geographic populations could be clearly separated using LDA by Bray-Curtis distance (Adonis, *R* = 0.105, *p* = 0.038, Figure 4C).

By using PICRUSt2, we predict the functional composition. Pathways level 1 includes metabolism, human diseases and cellular processes, and organismal systems. Pathways level 2 includes carbohydrate metabolism, drug resistance, endocrine, and infectious disease as the significant variation of predicted functional profiles of microbial communities among geographic populations (*p* < 0.01 or *p* < 0.05; see Table 2).

**Table 2:** The proportion of predicted functional profiles in different populations (KEGG Pathway level 1 and level 2).

	Yangjiang		Shantou		Xiamen		Fuzhou		Wenzhou		Ningbo	
	Mean	SD	Mean	SD	Mean	SD	Mean	SD	Mean	SD	Mean	SD
Pathway_Level _1												
Brite Hierarchies**	34.62%	1.23%	34.37%	0.92%	35.15%	1.31%	35.31%	0.96%	36.01%	0.49%	35.83%	0.59%
Metabolism**	35.62%	1.82%	36.18%	1.42%	34.62%	1.81%	35.17%	1.53%	33.65%	0.90%	33.87%	1.06%
Human Diseases**	3.27%	0.13%	3.20%	0.16%	3.52%	0.18%	3.39%	0.24%	3.38%	0.24%	3.36%	0.18%
Cellular Processes*	4.43%	0.38%	4.37%	0.33%	4.63%	0.44%	4.02%	0.25%	4.49%	0.34%	4.38%	0.56%
Organismal Systems*	1.85%	0.09%	1.88%	0.10%	1.78%	0.15%	1.80%	0.15%	1.70%	0.11%	1.67%	0.17%
Environmental Information Processing	6.06%	0.35%	6.16%	0.32%	6.24%	0.51%	6.23%	0.63%	6.35%	0.69%	6.77%	0.73%
Genetic Information Processing	6.80%	0.55%	6.55%	0.32%	6.55%	0.54%	6.47%	0.54%	6.55%	0.79%	6.32%	1.07%
Not Included in Pathway or Brite	7.37%	0.26%	7.29%	0.23%	7.52%	0.41%	7.61%	0.64%	7.86%	0.64%	7.79%	0.69%
Pathway_Level _2												
Carbohydrate metabolism**	9.38%	0.33%	9.64%	0.43%	8.97%	0.36%	9.66%	0.45%	9.10%	0.34%	9.48%	0.40%
Drug resistance: antimicrobial**	0.76%	0.09%	0.76%	0.08%	0.85%	0.11%	0.91%	0.14%	0.96%	0.13%	0.92%	0.17%
Endocrine system**	0.66%	0.04%	0.68%	0.05%	0.57%	0.09%	0.62%	0.08%	0.56%	0.07%	0.60%	0.11%
Infectious disease: bacterial**	0.68%	0.08%	0.65%	0.08%	0.79%	0.10%	0.64%	0.07%	0.75%	0.10%	0.72%	0.08%
Infectious disease: viral**	0.15%	0.04%	0.13%	0.04%	0.19%	0.04%	0.10%	0.03%	0.11%	0.05%	0.13%	0.07%
Protein families: metabolism**	5.83%	0.16%	5.72%	0.21%	6.01%	0.29%	5.96%	0.22%	6.04%	0.15%	5.86%	0.10%
Signaling molecules and interaction**	0.00%	0.00%	0.00%	0.00%	0.00%	0.00%	0.00%	0.00%	0.00%	0.00%	0.00%	0.00%
Transport and catabolism**	0.23%	0.05%	0.27%	0.04%	0.20%	0.05%	0.25%	0.08%	0.19%	0.05%	0.20%	0.04%
Cellular community – eukaryotes**	0.00%	0.00%	0.00%	0.00%	0.00%	0.00%	0.00%	0.00%	0.00%	0.00%	0.00%	0.00%
Substance dependence**	0.02%	0.01%	0.02%	0.01%	0.01%	0.02%	0.01%	0.01%	0.00%	0.00%	0.01%	0.01%
Metabolism of cofactors and vitamins*	4.05%	0.11%	3.94%	0.14%	4.06%	0.22%	3.84%	0.23%	3.93%	0.25%	3.65%	0.37%
Amino acid metabolism*	6.66%	0.73%	6.81%	0.55%	6.28%	0.76%	6.30%	0.48%	5.91%	0.36%	5.99%	0.59%
Lipid metabolism*	1.97%	0.26%	1.99%	0.20%	1.80%	0.31%	1.92%	0.15%	1.73%	0.09%	1.84%	0.20%
Development and regeneration*	0.02%	0.01%	0.02%	0.01%	0.02%	0.01%	0.02%	0.01%	0.02%	0.01%	0.01%	0.01%
Excretory system*	0.03%	0.00%	0.03%	0.01%	0.03%	0.01%	0.03%	0.01%	0.03%	0.01%	0.02%	0.01%
Metabolism of terpenoids and polyketides*	0.92%	0.12%	0.92%	0.10%	0.83%	0.15%	0.87%	0.08%	0.78%	0.07%	0.81%	0.11%
Unclassified: genetic information processing*	0.75%	0.07%	0.72%	0.05%	0.80%	0.07%	0.81%	0.13%	0.85%	0.15%	0.90%	0.19%

* Significant values as *p* < 0.05.

** Significant values as *p* < 0.01 by using non-parametric Kruskal–Wallis test.

### Latitude-associated alteration in gut microbiota

**Figure 5:**
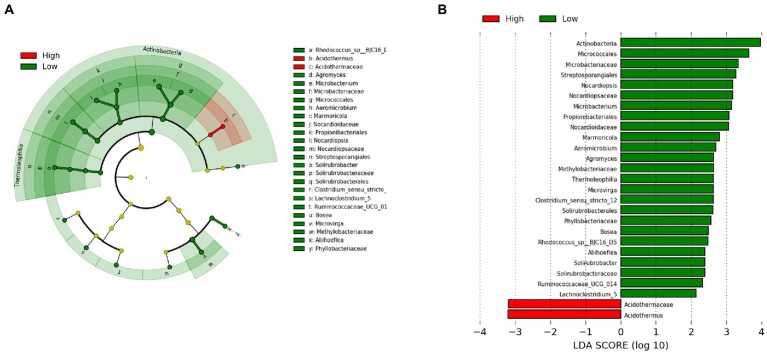
The taxa whose abundance differed between high latitudinal individuals (Wenzhou and Ningbo) and low latitudinal individuals (Yangjiang and Shantou) were identified by LDA effect size (LEfSe). **(A)** Taxonomic cladogram obtained from LEfSe analysis of sequences (relative abundance ≥0.5%). Biomarker taxa are highlighted by colored circles and shaded areas (high latitudinal samples are shown in red and low latitudinal samples are shown in green). Each circle’s diameter reflects the abundance of that taxa in the community. **(B)** The taxa whose abundance differed between the high latitudinal samples (red bars) and the low latitudinal samples (green bars). The cutoff value of ≥2.0 used for the LDA is shown.

To identify the specific bacterial taxa associated with latitude, we compared the gut microbiota of high latitudinal individuals (Wenzhou and Ningbo) and low latitudinal individuals (Yangjiang and Shantou), using the LDA effect size (LEfSe) method. A cladogram representative of the structure of the gut microbiota and the predominant bacteria is shown in Figures 5A,B, which also displays the greatest differences (LDA > 2) in taxa.

**Figure 6:**
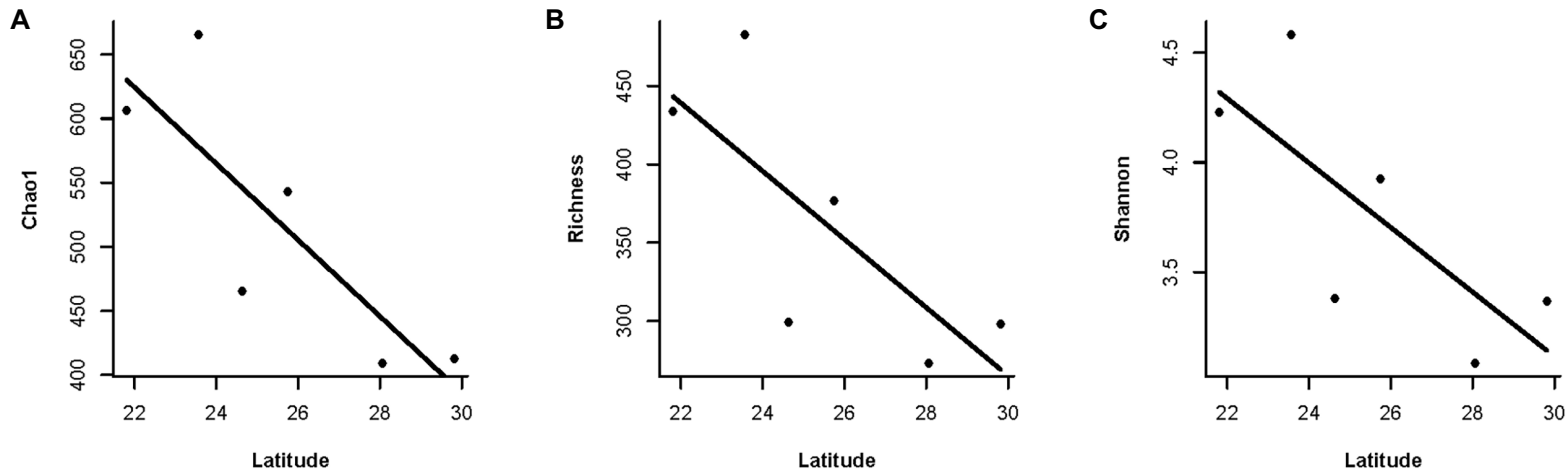
The relationship between latitude and gut microbial diversity along the eastern coast of mainland China. **(A)** Chao1 index. **(B)** Richness index. **(C)** Shannon index.

Moreover, we found a clear latitudinal pattern of gut microbial diversity in *F. limnocharis* populations on the eastern coast of mainland China (Figure 6, Supplementary Table S1). Gut microbial diversity of *F. limnocharis* decreased with increased latitude (*r* = −0.829, *p* < 0.05 for Chao1, Richness and Shannon). Furthermore, a positive correlation was found between the gut microbial diversity of *F. limnocharis* and the mean annual temperature (*r* = 0.829, *p* < 0.05 for Chao1, Richness and Shannon). Gut microbial diversity of *F. limnocharis* was not correlated to mean annual precipitation (*r* = −0.086, *p* = 0.872 for Chao1, Richness and Shannon) across sampling sites. Meanwhile, geographic distance had no significant influence on the gut bacterial beta-diversity of *F. limnocharis* (Mantel test: *p* > 0.05) (Supplementary Table S2).

## Discussion

We studied variations in the *F. limnocharis* gut microbiota sampled across a large latitudinal range along the eastern coast of mainland China. Our results showed that the composition of the “important” gut microbiota of *F. limnocharis* had no significant difference between our study and studies in other parts of China. Importantly, we found several biogeographic general patterns. First, a significant correlation between gut microbial diversity (alpha-diversity) and latitude was observed in our research system. Second, we discovered that latitude influenced the composition of gut microbiota (beta-diversity). Finally, we detected that geographical distance could not determine the gut microbiota composition in *F. limnocharis*. In conclusion, we determined the most significant contributing factor to the microbial diversity of *F. limnocharis* among populations along the eastern coast of mainland China was their geographic latitude.

We observed significant negative correlations between alpha-diversity and latitudinal gradient. This result supports the hypothesis that species diversity is negatively correlated with latitude (Schemske et al., 2009). Host associated microbiomes display similar patterns was rarely found in natural populations (Meyer et al., 2018; Ge et al., 2021). For example, Ge et al. (2021) found that two honeybee species (*Apis cerana* and *Apis mellifera*) presented similar patterns that population of low latitudes harbored gut bacterial communities with a higher diversity in five geographically distant sites along a latitudinal gradient (Ge et al., 2021). However, the negative correlation between leaf fungal endophyte communities and several tree species was detected in a broad latitudinal gradient from the Canadian arctic to the lowland tropical forest of central Panama (Arnold and Lutzoni, 2007). The inconsistent pattern was also discovered between wild house mice and gut microbial diversity from three latitudinal transects across North and South America (Suzuki et al., 2020). Furthermore, the neutral dynamics dominated the gut microbial diversity of fly (*Drosophila melanogaster*) along a latitudinal cline (Henry and Ayroles, 2022). Compared with macro-organisms, microbial biogeographic patterns tend to be much weaker (Meyer et al., 2018; Henry and Ayroles, 2022). Longevity, or dispersal abilities of the microbiome are a possible explanation for this phenomenon (Meyer et al., 2018). However, *F. limnocharis* have the shortness of life (about 3–5 years for males and 4–5 years for females) and poor dispersal ability (Li et al., 2011). Our results clearly showed that geographic latitude has contributed to the gut microbial diversity pattern of *F. limnocharis* populations along the eastern coast of mainland China.

We found that latitude may explain significant differences in microbial variation in our research system. Latitude might be viewed as an approximation for the sum of environmental effects such as local weather patterns, which incorporates average annual temperatures and precipitation. In this study, we distinguish the effects of these ecological factors. We detected that gut microbial diversity of *F. limnocharis* was positively associated with mean annual temperature. This result is similar to that found in previous studies (Tsuchida et al., 2002; Huang and Zhang, 2013; Ge et al., 2021). However, we could not determine the relationship between gut microbial diversity with mean annual precipitation. One possible explanation for this is that there is too much rainfall along the eastern coast of mainland China (Shi et al., 2015).

Several studies have suggested that host genetics impacts composition of ectotherm gut microbial communities (Yuan et al., 2015; Shu et al., 2019; Xu et al., 2020; Chen et al., 2022). They have reported various dietary effects on ectotherm gut microbiota across different environments (Kohl et al., 2014; Chang et al., 2016; Knutie et al., 2017; Tong et al., 2019; Montoya-Ciriaco et al., 2020; Tang et al., 2020; Wang et al., 2021). While we cannot rule out the possible contribution of host genetics or dietary availability to the gut microbial variation of *F. limnocharis* in the eastern coastline of mainland China, we sampled content microbial communities in a rigorous context. First, the collected samples were males and females of equal weight, in the same seasons, and from different sampling sites, respectively. This is due to fact that dietary variations and the body mass of *F. limnocharis* were significantly correlated with gut microbial composition, and the composition of gut microbiota of *F. limnocharis* vary seasonally in response to diet variations (Chang et al., 2016; Huang and Liao, 2021). Second, similar bacteria were found as in other studies of natural *F. limnocharis* populations (Chang et al., 2016; Xu et al., 2020).

In summary, we found that there was a significant variation in the gut microbiota diversity of *F. limnocharis* along the eastern coast of mainland China, and latitude had a significant effect on the gut microbial composition of natural populations of *F. limnocharis*. However, our understanding of the importance of latitude for the gut microbial communities of animals was limited because samples were only collected from shoreline frog populations. Thus, further studies (such as longitudinal sampling or controlled transplant experiments of microbes) are needed in order to comprehensively understand the composition, structure, and function of the gut microbiome of *F. limnocharis* in natural populations (Suzuki et al., 2020; Henry and Ayroles, 2022). Our study provided the first evidence that latitude might shape the gut microbiota of frog along the eastern coast of mainland China.

## Data availability statement

The original contributions presented in the study are included in the article/Supplementary material, further inquiries can be directed to the corresponding authors.

## Ethics statement

The animal study was reviewed and approved by Anhui Normal University.

## Author contributions

SW, SL and NZ designed the study and wrote manuscript. NZ, ZM, YJ, YS, YX, and YW collected samples. NZ, ZM, YJ, YS, SW, YX, and YW performed experiments. NZ and SW analyzed data. All authors contributed to the article and approved the submitted version.

## Conflict of interest

The authors declare that the research was conducted in the absence of any commercial or financial relationships that could be construed as a potential conflict of interest.

## Publisher’s note

All claims expressed in this article are solely those of the authors and do not necessarily represent those of their affiliated organizations, or those of the publisher, the editors and the reviewers. Any product that may be evaluated in this article, or claim that may be made by its manufacturer, is not guaranteed or endorsed by the publisher.
