# Supplementary material for: Geographical patterns of Fejervarya limnocharis gut microbiota by latitude along mainland China’s coastline
Source: Front Microbiol. 2022 Nov 17;13:1062302. doi: 10.3389/fmicb.2022.1062302 (PMC9713514; doi:10.3389/fmicb.2022.1062302)
Supplement: Supplementary file 1 [file Data_Sheet_1.PDF]

Supplementary Files (two figures and two tables) for

Geographical patterns of *Fejervarya limnocharis* gut microbiota by latitude along mainland China's coastline

Na Zhao [1,2,3], Zhiwei Ma[1], Yixin Jiang[4], Yingying Shi[4], Yuning Xie[4], Yuting Wang[4], Siyu Wu[4], Shelan Liu[5*] and Supen Wang[4*]

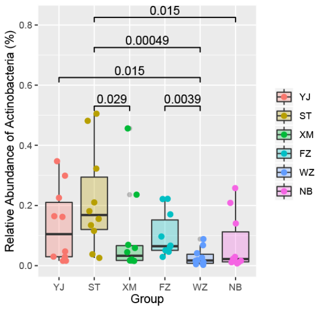

A) Phylum

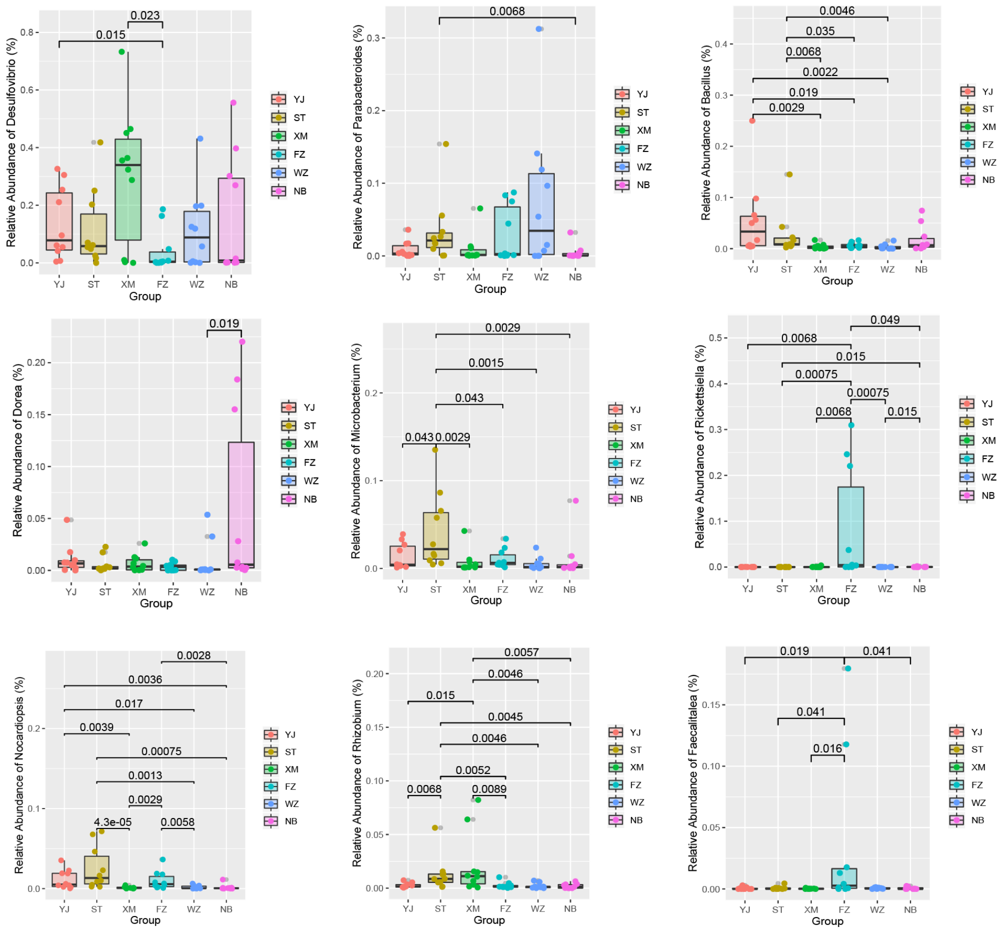

B) Genus

Supplementary Figure 1. The abundance diversity differed significantly among population in Phylum and Genus level by using wilcox test.
A) Phylum level. B) Genus level.

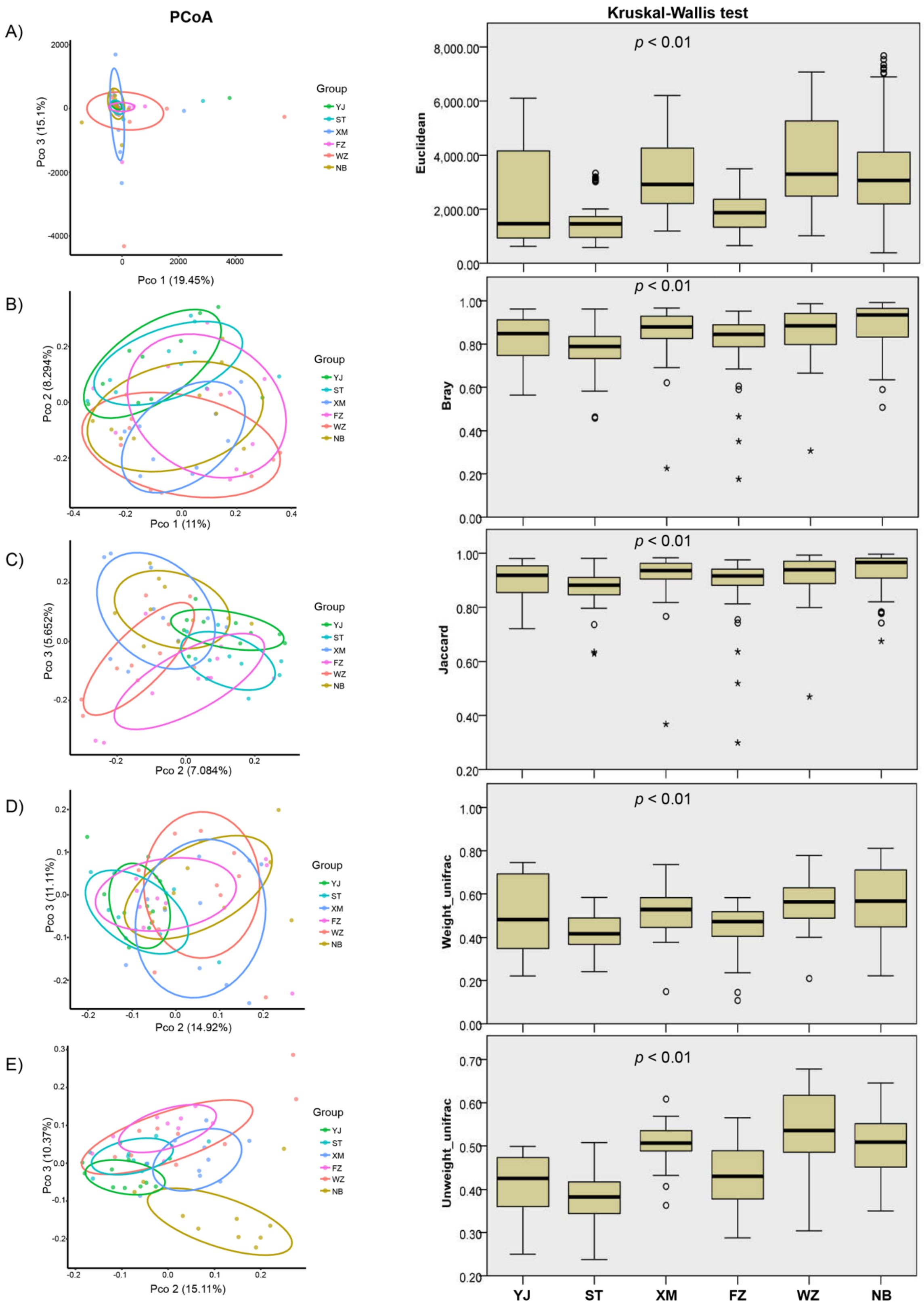

Supplementary Figure 2. The variation of bacterial diversity of the gut microbiota.

A) Principal coordinates analysis (PCoA) among different populations. B) The composition analysis by using Kruskal-Wallis test.

Supplementary Table 1. The correlation among Alpha-diversity of *F. limnocharis* gut microbiota and climate factors and latitude and longitude

	Mean annual temperature	Annual precipitation	Latitude	Longitude
Chao1	.829*	-0.086	-.829*	-.829*
Richness	.829*	-0.086	-.829*	-.829*
Shannon	.829*	-0.086	-.829*	-.829*

Spearman test, * indicates $p < 0.05$.

Supplementary Table 2. The correlation between beta diversity and geographical distance by using Mantel test.

Beta diversity	r	*p*
Bray	0.26	0.23
Euclidean	0.05	0.47
Jaccard	0.26	0.23
Unweigh Unifrace	0.03	0.49
Weight Unifrace	0.31	0.20
